# Diffusion engineering of ions and charge carriers for stable efficient perovskite solar cells

**DOI:** 10.1038/ncomms15330

**Published:** 2017-06-12

**Authors:** Enbing Bi, Han Chen, Fengxian Xie, Yongzhen Wu, Wei Chen, Yanjie Su, Ashraful Islam, Michael Grätzel, Xudong Yang, Liyuan Han

**Affiliations:** 1 State Key Laboratory of Metal Matrix Composites, School of Materials Science and Engineering, Shanghai Jiao Tong University, Shanghai 200240, China; 2 Photovoltaic Materials Unit, National Institute for Materials Science, Tsukuba, Ibaraki 305–0047, Japan; 3 Key Laboratory for Thin Film and Microfabrication of the Ministry of Education, Department of Micro/Nano Electronics, School of Electronics, Information and Electrical Engineering, Shanghai Jiao Tong University, Shanghai 200240, China; 4 Laboratory of Photonics and Interfaces (LPI), Station 6, Institute of Chemical Science and Engineering, Faculty of Basic Science, Ecole Polytechnique Federale de Lausanne, CH-1015 Lausanne, Switzerland

## Abstract

Long-term stability is crucial for the future application of perovskite solar cells, a promising low-cost photovoltaic technology that has rapidly advanced in the recent years. Here, we designed a nanostructured carbon layer to suppress the diffusion of ions/molecules within perovskite solar cells, an important degradation process in the device. Furthermore, this nanocarbon layer benefited the diffusion of electron charge carriers to enable a high-energy conversion efficiency. Finally, the efficiency on a perovskite solar cell with an aperture area of 1.02 cm^2^, after a thermal aging test at 85 °C for over 500 h, or light soaking for 1,000 h, was stable of over 15% during the entire test. The present diffusion engineering of ions/molecules and photo generated charges paves a way to realizing long-term stable and highly efficient perovskite solar cells.

## Abstract (web summary)

Ion migration in perovskite solar cells are known to cause hysteresis and instability. Bi *et al*., report a charge extraction layer based on graphene, fullerenes and carbon quantum dots which suppresses ion diffusion and enhances charge carrier diffusion leading to efficient devices with improved stability.

Hybrid organic–inorganic perovskite materials are the subject of intense current investigations owing to their excellent photovoltaic properties, for example, intense visible (vis) light absorption, tuneable band gap and long charge carrier diffusion length^1^,^2^,^3^,^4^,^5^,^6^,^7^. The perovskite solar cells (PSCs) have been considered as the most competitive next generation photovoltaic technology that exhibits the advantages of low-cost processing, abundantly available materials and unprecedented rise in solar to electric power conversion efficiency (PCE) (refs 8, 9, 10, 11, 12, 13, 14). The state-of-the-art PSCs reached a PCE of 22% (ref. 14) and demonstrated the feasibility of achieving PCEs of over 25% for tandem cells^15^. However, the long-term stability of PSCs is notoriously poor, which has been considered as one of the major challenges for future large-scale application^16^,^17^,^18^,^19^.

The long-term stability of PSCs has been restricted owing to the low-energy barrier for ions/molecules migration within perovskite light absorbers whose crystal lattice can be easily changed or collapsed under thermal activation, light soaking, moisture invation^9^,^20^, photo-induced reaction^21^, or phase separation^22^,^23^ etc., Among the tremendous efforts to suppress the degradation, some approaches have successfully improved the device stability, including the introduction of carbon counter electrode^9^, composition engineering with mixed anions^11^ or cations^15^, heavily doped metal oxide as robust charge transporting materials^12^, surface passivation^24^, hydrophobic materials^25^ and crystal growth with reduced defects^26^ etc., However, it is still a big challenge to achieve highly efficient PSCs exhibiting long-term stable performance.

Here, we provide a strategy for high efficiency and long-term stability via diffusion engineering to hinder unfavourable ions/molecules diffusion and accelerate photo generated charges diffusion within PSCs. We deposited a nanostructured carbon layer acting as an ions/molecules blocking and electron extraction layer (EEL), containing N-doped graphene, the fullerene derivative phenyl-C61-butyric acid methyl ester (PCBM) and carbon quantum dots (CQDs), between the perovskite light absorber layer and the electrode layer. In comparison with conventional strategy of increasing the thickness of blocking layers, the present strategy enabled a higher capability of almost 3 times of that of conventional EELs in blocking ions/molecules diffusion at the same thickness. The major contribution was attributed to the graphene derivatives in the nanocarbon EEL. The layer-to-layer diffusion of iodide from iodide-rich perovskite layer to the electrode layer was successfully hindered for both of the CH_3_NH_3_ (methylammonium or MA) or HC(NH_2_)_2_ (formamidinium or FA) based perovskite materials, where such diffusion has been reported as a serious device degradation or defects generation process causing poor device stability^16^,^27^,^28^. This undesirable diffusion is related to the relatively low activation energy for iodide migration and the high iodide concentration gradient of about 10^26^ cm^−4^ within a tiny distance of tens of nanometers from the perovskite surface to the electrode, which can be further accelerated at a higher temperature^16^,^23^,^27^,^28^,^29^,^30^. The diffusion of Ag atoms from electrode to perovskite layer, another factor for the device degradation^31^, was also retarded by the present nanocarbon layer. In addition, the designed nanocarbon layer also exhibited a high electric conductivity and good ohmic contact with the metal electrode. As a result, we were able to obtain centimetre-squared device with a certified efficiency of 15.6% recorded in the solar cell efficiency tables^32^. Our work also presented the PSCs had good thermal stability and light stability, which exhibited a stable efficiency of over 15% during thermal aging test at 85 °C for 500 h or light soaking under air mass 1.5 global (AM 1.5G) illumination for 1,000 h. This strategy provides a promising way to the future practical application of highly stable and efficient large-area PSCs.

## Results

### Device structure

**Figure 1:**
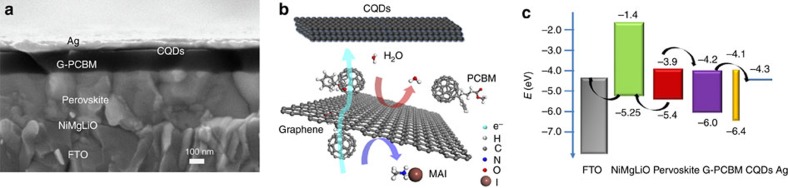
Planar heterojunction PSCs using a nanocarbon layer to suppress layer-to-layer ions/molecules diffusion and facilitate electron charge carrier diffusion. (**a**) SEM cross-section image of a NiMgLiO/Perovskite/N-doped graphene fullerene derivative phenyl-C61-butyric acid methyl ester (G-PCBM)/CQDs/Ag device, in which G-PCBM is graphene doped PCBM. (**b**) Schematic drawing of the diffusion processes within the nanocarbon-based EEL. (**c**) Energy band structure of the planar heterojunction cell.

We prepared PSCs with a planar heterojunction structure of layers superimposed in the sequence FTO/NiMgLiO (20 nm)/MAPbI_3_ (350 nm)/G-PCBM (50–200 nm)/CQDs (10 nm)/Ag (100 nm) (Fig. 1a). G-PCBM stands for PCBM doped with 2 wt% N-doped graphene to block the layer-to-layer diffusion of iodide or water molecules (Fig. 1b). The work function for CQDs was derived from UPS measurements (Supplementary Fig. 1) showing the conduction band of CQDs to be positioned at –4.1 eV (Fig. 1c).

### Iodide diffusion

To scrutinize the influence of the nanocarbon layer on the layer-to-layer diffusion of iodide, we compared the UV–vis absorption spectra of MAPbI_3_ perovskite films coated with different nanocarbon layers (Supplementary Fig. 2) and used the light absorbance at 600 nm to assess the fraction of the perovskite materials that remained intact. All samples were put on a hotplate at 100 °C in N_2_ for thermal aging test to accelerate the diffusion process. The perovskite film exposed to N_2_ was almost decomposed within 15 h under thermal heating. The reason for the thermal decomposition of perovskite film is that the activation barrier of migration of iodide to its vacancy is relatively small^29^,^30^. The perovskite film can be thermally decomposed to PbI_2_ and MA, and HI, where the MA and HI can escape from the perovskite surface and left iodide vacancies. At a higher temperature, the iodide has a higher energy to enable a faster diffusion from the inside of the perovskite film to the surface vacancies. With the increase of vacancy defects at the surface, the movement of iodide within the perovskite film and the consequent degradation will be accelerated. In contrast, for the MAPbI_3_ film covered with 150 nm G-PCBM, the light absorbance at 600 nm lost only 8% after 180 ageing hours, which was almost 3 times longer than that of the samples covered with PCBM film at the same thickness. The thermal stability of (FAPbI_3_)_0.85_(MAPbBr_3_)_0.15_ perovskite film was also greatly enhanced when covered with the G-PCBM film (Supplementary Fig. 3). These results strongly suggest that the graphene derivatives in the present nanocarbon layer can effectively hinder the loss of iodide from the perovskite film and the consequent degradation in comparison with conventional PCBM EELs.

**Figure 2:**
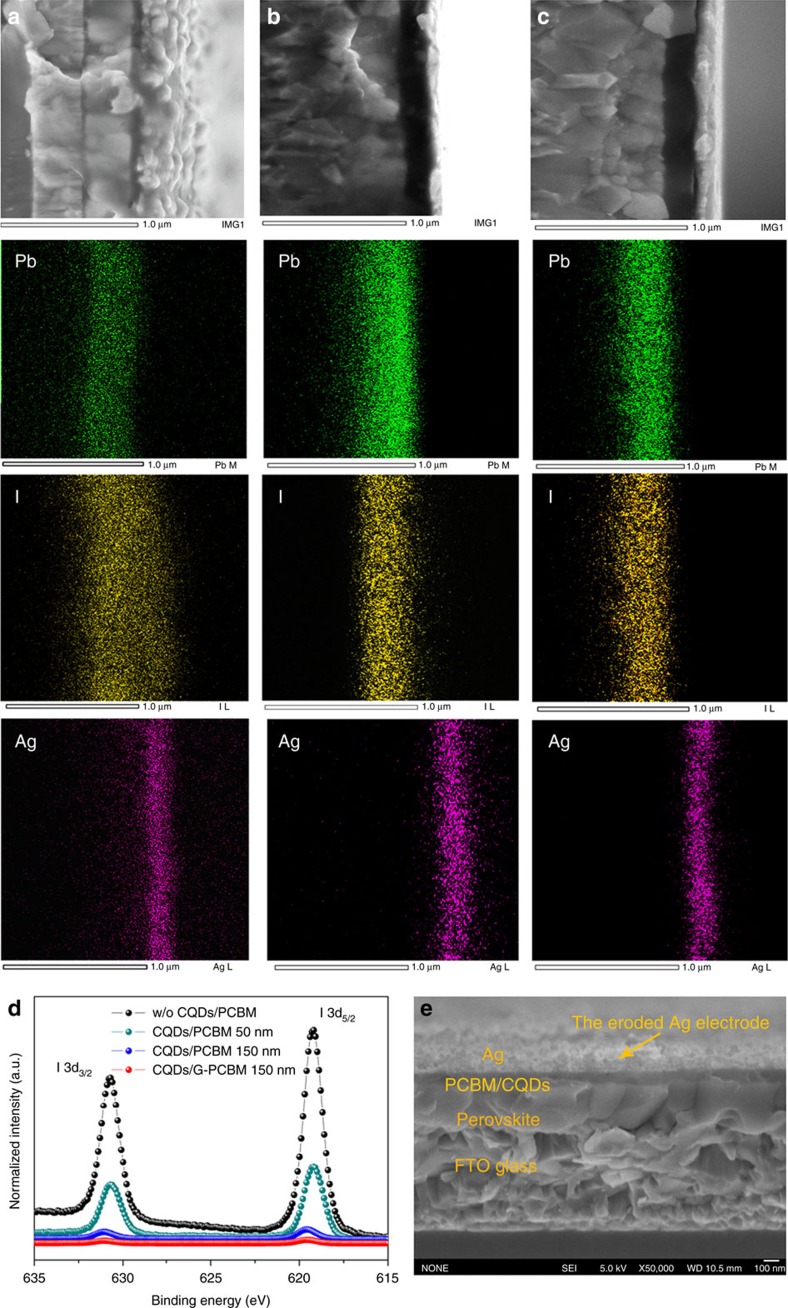
Monitoring iodide distribution in PSCs. (**a**–**c**) SEM-EDX analysis of PSCs based on CQDs/fullerene derivative phenyl-C61-butyric acid methyl ester (PCBM) (50 nm), CQDs/PCBM (150 nm) and CQDs/N-doped graphene fullerene G-PCBM (150 nm), respectively. The PSCs were preheated at temperature of 100 °C in the dark and dry conditions for 90 h. (**d**) XPS spectra of iodide at silver electrode. (**e**) SEM image of cross-section of PCBM (50 nm) based device after thermal aging in the ambient environment for 7 days.

As the diffused iodide across the EEL could corrode the silver electrode, we further determined the spatial distribution of iodide in thermally aged PSCs. The scanning electron microscopy with energy dispersive X-ray (SEM-EDX) analysis (Fig. 2a–c) indicate that the iodide diffused across the EEL to the silver electrode in 50 nm PCBM based device while it was almost hindered in 150 nm PCBM or G-PCBM based device. In Fig. 2d, the X-ray photoelectron spectroscopy (XPS) spectra shows the signal from iodide in silver electrode of the 150 nm G-PCBM based device was the smallest and only one-third of that of the 150 nm PCBM based device, indicating the graphene derivatives blocking the iodide diffusion effectively. In addition, the silver electrode was smooth than the rough one in the 50 nm PCBM based devices that was caused by the iodide corrosion (Fig. 2a,e). We note that the iodide, with ions diameter of 0.412 nm, cannot penetrate the two-dimensional crystal lattice of graphene (lattice parameter *a*=0.246 nm). So in a G-PCBM layer, the diffused iodide has to move round the graphene derivatives (the typical size is of about 100 nm). The total diffusion path of iodide or other molecules in G-PCBM EEL with a typical thickness of 150 nm would be extended about 10 times than that of 50 nm PCBM EEL. According to Fick's law, a long diffusion length can reduce the concentration gradient and consequently the diffusion flux, which can hinder the diffusion of iodide out from the perovskite film and consequent decomposition. Another possible reason for the decomposition of perovskite film is the diffusion of Ag into perovskite film^31^. The Ag diffusion would be enhanced if the nanocarbon layer cannot fully cover the surface of perovskite film that may make perovskite/Ag contact. We used a solvent engineering method to deposit smooth perovskite film with roughness lower than 20 nm so that the nanocarbon layer with thickness of 50 nm or larger can fully cover the perovskite films^33^. As shown in Supplementary Fig. 4, the perovskite film covered with Ag electrode, in the perovskite/PCBM (150 nm)/CQDs/Ag cell, lost the typical dark-brown colour and turned to be yellow while the perovskite film without Ag showed less change, which indicate the perovskite degraded faster than that without Ag electrode. In comparison, the perovskite film covered with G-PCBM (150 nm)/CQDs/Ag film was much stable with negligible change after the same ageing test. This result strongly suggests that the present nanocarbon layer is desired as a more effective barrier to prevent direct perovskite/Ag contact and suppress Ag or iodide diffusion from layer-to-layer.

### Device stability

**Figure 3:**
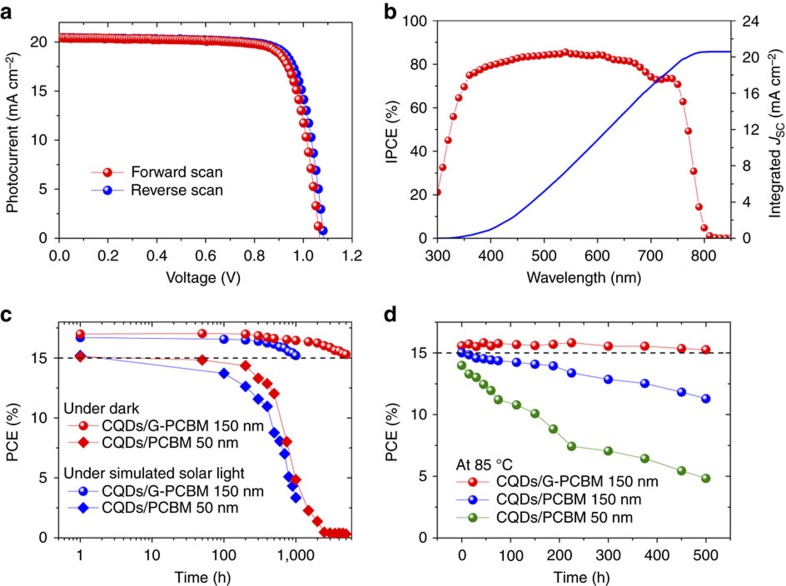
Performance of PSCs. (**a**) The photocurrent current–voltage characteristics of one device with the structure of NiMgLiO/Perovskite/N-doped graphene fullerene derivative phenyl-C61-butyric acid methyl ester (G-PCBM) (150 nm)/CQDs (10 nm)/Ag, measured under simulated solar light (AM 1.5G, 100 mW cm^−2^). (**b**) Its IPCE spectrum. (**c**) The stability of sealed devices under dark and under air AM 1.5G simulated solar light. (**d**) The stability of sealed cells in thermal ageing test at 85 °C in an atmosphere with relative humidity of about 50%.

The device stability was then studied to evaluate the effects of different nanocarbon layers. As shown in Fig. 3a, the best of the optimized devices using CQD/G-PCBM (150 nm) exhibited a small hysteresis in current–voltage (*J–V*) characteristics with a PCE of 17.0% at forward scan and 17.4% at reverse scan under simulated AM 1.5G solar light with an aperture area of 1.02 cm^2^ (Supplementary Fig. 5). This device performed stably during a short-term test of 200 s (Supplementary Fig. 6). We measured the incident photon-to-current conversion efficiency (IPCE) spectrum (Fig. 3b) and the integrated *J*_SC_ matched well with the value in *J–V* characteristics. For the long-term stability test of sealed cells, the PCE of a CQDs/G-PCBM (150 nm) based device was stable of over 15% when the device had been kept under dark at room temperature for 5,000 h or under AM 1.5G simulated solar light for 1,000 h (Fig. 3c). Particularly, during the thermal ageing test at 85 °C for 500 h, the PCE was also stable of over 15% with remaining 98% of the initial value (Fig. 3d and Supplementary Fig. 7). In contrast, the device with the same thickness PCBM layer showed obvious degradation with a loss of 20% of the initial PCE, where a worse light absorbance of a decomposed perovskite film reduced the photocurrent density and a worse Ag electrode lowered the fill factor. We highlighted the stable efficient PSCs with an efficiency of over 15% during long-term thermal ageing and light soaking test, which indicates our designed nanocarbon layer plays the key role in the device stability.

The unsealed devices with CQDs/G-PCBM layer also enabled a better stability than other ones (Supplementary Fig. 8a). We further compared unsealed devices using CQDs, LiF (the work function is 3.8 eV) or Ca (the work function is 2.9 eV) as the interlayer between PCBM and silver. The PCE of LiF or Ca based devices dropped more than 30% or 60% due to their sensitivity to air and water. By contrast, the CQDs based devices showed less degradation of 17% than LiF and Ca based devices during ageing test (Supplementary Fig. 8b). Therefore, the CQDs/G-PCBM layer can suppress the diffusion of iodide escaping from the iodide-rich perovskite layer and the diffusion of molecules from air into the device, which is a key advantage for the realization of stable PSCs by suppressing the generation of defects.

The effect of layer thickness of CQDs/PCBM and CQDs/G-PCBM on the device performance were studied (Supplementary Fig. 9, Supplementary Tables 1 and 2). Although CQDs/PCBM (lower than 50 nm) based devices showed high performance with PCE more than 15%, the efficiencies varied over a wide range (Supplementary Fig. 10) indicating poor reproducibility in producing 1-cm^2^-sized PSCs. The devices with CQDs/PCBM films showed better reproducibility, however, the PCEs were low because of the loss in FF and *J*_SC_. On the contrary, the PCEs of CQDs/G-PCBM devices were relatively insensitive to its thickness, implying a good charge transport within the CQDs/G-PCBM layer.

We then compared the electronic conductivity of PCBM and G-PCBM films by scanning probe microscope (Supplementary Fig. 11). At a bias potential of 1 V, the average of electric current through the 150 nm G-PCBM EEL was 23.1 nA, which was 5.6 times as large as that of 4.1 nA for the PCBM reference of the same thickness. These films also exhibited trap-free space charge limit current (SCLC) in the bias range of 0.1–1.0 V (Supplementary Fig. 11e). Using the Mott–Gurney law and Einstein relation to express the voltage dependence of the current density:


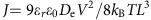


where *J* is current density, *ɛ*_*r*_ is relative dielectric constant of PCBM and *ɛ*_0_ is the vacuum permittivity, *D*_e_ is the electron diffusion coefficient, *V* is applied voltage, *k*_B_ is the Boltzmann constant, *T* is the temperature in kelvin and *L* is the thickness of the layer, we derived electron diffusion coefficients, of 7.80 × 10^−5^ cm^2^ s^−1^ and 1.48 × 10^−5^ cm^2^ s^−1^ for the G-PCBM and PCBM films respectively. This agrees well with the results obtained from SPM and follows the observed trends in device performance (Supplementary Fig. 9c). For pristine PCBM films, the phase image clearly showed the aggregates of PCBM with the size ranging from 20 to 50 nm (Supplementary Fig. 12). The values of *R*_ms_ of PCBM film and G-PCBM were 6 and 1 nm, respectively, both of which were much less than the preferred thickness of 150 nm. The slightly improved morphology may also contribute a little to suppress the ions migration. Thus, from the phase and surface topology of the PCBM and G-PCBM films, we infer that the G-PCBM EEL formed a compact layer which assists electron transport while impairing penetration of ions or water.

**Figure 4:**
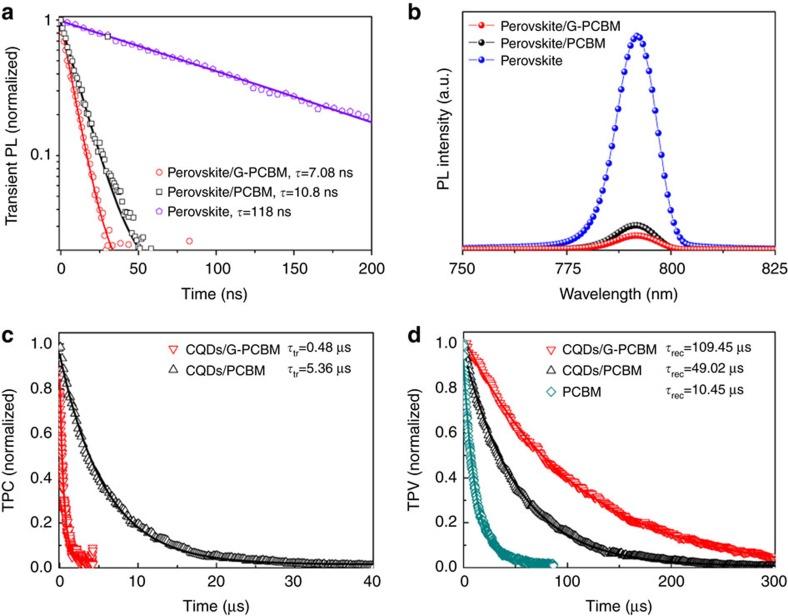
Electron extraction and charge recombination properties. (**a**,**b**) TRPL decay curves and steady-state PL of MAPbI_3_ films covered by fullerene derivative phenyl-C61-butyric acid methyl ester (PCBM) and N-doped graphene G-PCBM. Solid lines are fitted results with a double exponential decay. (**c**,**d**) The transient photocurrent and photovoltage decay curves of MAPbI_3_ devices with CQDs/PCBM and CQDs/G-PCBM, respectively.

We evaluated the electron extraction dynamics via steady-state photoluminescence (PL) and time-resolved photoluminescence (TRPL) measurements (Fig. 4a,b). For the MAPbI_3_ film alone, a long lifetime of more than 100 ns was observed. This suffices to ensure a carrier diffusion length of hundreds of nanometers, comparable to the thickness of the light harvesting film^4^. The PL lifetime for the perovskite/PCBM film was 10.8 ns, which is consisted with a previous report^28^. The perovskite/G-PCBM showed the PL lifetime of being shortened to 7.08 ns, indicating a faster charge extraction. Correspondingly, a strong quenching of the steady-state PL was observed for the perovskite/G-PCBM film than perovskite or perovskite/PCBM film. The electron capture is likely to be accelerated by the faster electron transport in the G-PCBM compared to the reference PCBM film^34^,^35^.

The charge transport and recombination time constants (*τ*_tr_ and *τ*_rec_) were derived from the transient photocurrent and voltage measurements performed under short-circuit and open-circuit conditions, respectively (Fig. 4c,d). The CQDs/G-PCBM based devices exhibited a much more rapid photocurrent decay (*τ*_tr_ of about 0.48 μs) than that of the CQDs/PCBM (*τ*_tr_ of about 5.36 μs), confirming the faster electron transport in the CQDs/G-PCBM film. This was in good agreement with our observation that the electron diffusion coefficient in G-PCBM was about 5.3 times larger than that in PCBM. The CQDs/G-PCBM (150 nm) device with a *V*_OC_ of 1.09 V exhibited slower photovoltage decay (*τ*_rec_ of about 109.45 μs), indicating a better retardation of carrier recombination compared to that of a CQDs/PCBM (150 nm) device (*τ*_rec_ of about 49.02 μs, *V*_OC_ of 1.05 V). We also compared the recombination properties of a CQDs/PCBM with the PCBM reference. The photovoltage decay time in an aged device with a neat 50 nm PCBM film (with stable *V*_OC_ of 0.86 V) was 10.45 μs, which is much shorter than that observed in CQDs/PCBM device. Thus, the present nanocarbon layer contributed to the suppression of the unwanted carrier recombination process. As a result of the improvement in charge transport and recombination, CQDs/G-PCBM devices exhibited superior PCEs than CQDs/PCBM devices at the same thickness ranged from 50 to 200 nm.

We also investigated the effect of the CQD layer thickness on the PV metrics and present data in Supplementary Table 3. The PCE was improved up to 15.8% for devices with CQDs thickness of 10 nm, much larger than that of 8.1% for a CQD-free device. As shown in Supplementary Fig. 8d, the S-shaped *J–V* curves of CQD-free cells in the forward bias range from 0.6 to 1.0 V indicated the formation of a Schottky-barrier at the PCBM/Ag junction^36^. The application of CQDs buffer layer rectified the shape of *J–V* curves and improved both the *FF* and *J*_SC_ (Supplementary Table 4). Hence apart from retarding charge carrier recombination, the CQDs eliminated the Schottky-barrier at the junction of PCBM/Ag by forming an ohmic contact that facilitated the electron transport through the interface. We sent one of our cells to a public test centre for certification, which obtained a PCE of 15.6% (Supplementary Fig. 13). This was also recorded for certified PSCs with an aperture area of more than 1.0 cm^2^ (ref. 24).

## Discussion

In summary, we demonstrated an approach for highly stable efficient PSCs by controlling the diffusion process of charge carriers and ions/molecules within PSCs with a nanocarbon layer consisting of graphene derivatives, fullerene derivatives and CQDs. The ions/molecules diffusion was effectively hindered by 3 times in the graphene-PCBM EEL in comparison with that of conventional PCBM EEL. The device stability was greatly improved owing to the retardation of ions/molecules diffusion. Meanwhile, the diffusion of electron charge carriers was enhanced to enable a high FF for the device. This work indicates the importance of diffusion engineering for future application of stable and efficient PSCs.

## Methods

### Device fabrication

The FTO substrate was first patterned by etching with a 2 M HCl solution and Zn powders around a mask formed by strips of adhesive tape. Then, 30 ml of an acetonitrile/ethanol (with 95:5 volume ratio) solution of nickel acetylacetonate (with 15 mol% magnesium acetate tetrahydrate and 5 mol% lithium acetate) was sprayed by an air nozzle onto the hot FTO glasses at 570 °C. A dense hole transfer layer of NiMgLiO (about 20 nm in thickness) was prepared onto the FTO substrate^12^. The prepared MAI and PbI_2_ for 1.45 M CH_3_NH_3_PbI_3_ solution were stirred in DMSO at 70 °C for 12 h. The resulting solution was coated onto the NiMgLiO/FTO substrate by a consecutive spin-coating process at 1,000 and 5,000 r.p.m. for 10 and 20 s, respectively. During the second spin-coating step, the perovskite film was dripped with 1 ml toluene drop-casting. The substrate was thermal treatment on a hotplate at 100 °C for 10 min. A solution of G-PCBM (40 mg ml^−1^ in chlorobenzene) with 2 wt% graphene was dissolved at 85 °C by stirring for 12 h before use. The synthesis method of functionalized graphene and CQDs were supplied in the Supplementary Information. The G-PCBM with different thickness was deposited on the perovskite/NiMgLiO/FTO substrate by various spin-coating rates for 30 s, with 3,500, 2,000, 1,500 and 800 r.p.m. spin-coat rates for 50, 100, 150 and 200 nm G-PCBM, respectively). In comparison, the PCBM film without graphene addition was also prepared on perovskite/NiMgLiO/FTO substrate. After the film was dried at 70 °C for 10 min, the interfacial layer was prepared from 0.2 mg ml^−1^ CQDs methanol solution dripping-coating on G-PCBM or PCBM/perovskite/NiMgLiO/FTO substrate at 5,000 r.p.m. for 60 s. The film was dried at 70 °C for another 10 min. At last, one batch of films was transferred to the evaporator chamber, 100 nm Ag or Au/Ag was deposited under high vacuum (lower than 2 × 10^*−*4^ Pa). The solar cell was sealed by UV glue coating on a cavity glass which covered between the front FTO glass and the active films. All of these processes were done in glove box.

### Characterization and measurement

Morphologies of as-obtained products were observed on a field emission scanning electron microscope (SEM, JEOL JSM-6500F). Transmission electron microscopy (TEM, JEOL JEM-2100F) images were obtained under an acceleration voltage of 200 kV. UV–vis absorption spectra were recorded on a Shimadzu UV 2550 spectrophotometer in the 200–800 nm wavelength range at room temperature. AFM was performed using Bruker multimode 8 in ‘tapping' mode. The crystal structures of perovskite film were characterized by powder X-ray diffraction using a Goniometer Ultima IV (185 nm) diffractometre with Cu K_α_ radiation, excited at 40 kV and 40 mA. Raman spectra were taken on a DXR Raman Microscope with an excitation length of 532 nm.

Photovoltaic measurement employed a black metal mask with an aperture area of 1.02 cm^2^ under standard air mass 1.5G sunlight (100 mW cm^−2^, WXS-155S-10: Wacom Denso Co., Japan) and the simulated light intensity was calibrated with a silicon photodiode. The light soaking stability was tested in a solar cell light resistance test system (Model BIR*-*50, Bunkoh-Keiki Co., LTD) equipped with a Class AAA solar simulator; the UV light less than 420 nm was cut off with an optical filter. *J–V* curves of the PSCs were measured with a digital source metre (Keithley 2400) under simulated solar illumination at 100 mW cm^–2^, AM 1.5G standard and a calibrated Si-reference cell. The *J*–*V* curves were measured by reverse (from 1.2 to −0.2 V) or forward (from −0.2 to 1.2 V) scan. The step voltage was fixed at 10 mV and the delay time with 50 ms. Monochromatic IPCE spectra were measured with a monochromatic incident light of 1 × 10^16^ photons cm^−2^ in director current mode (CEP-2000BX, Bunko-Keiki).

The TRPL characterizations were measured on an Edinburg FLS 920 (Edinburg 90 Co. LTD), and the excitation was at the wavelength of 445 nm with pulse width of 95.3 ps provided by a picosecond pulsed light emitting diode (EPLED-445). Transient photovoltage/photocurrent decay curves were measured on a home-made system with a white light bias generated from an array of diodes (100 mW cm^−2^) and red light pulse diodes (0.05 s square pulse width, 100 ns rise and fall time, 5 mW cm^−2^) controlled by a fast solid–state switch were used as the perturbation source. The transient photocurrent was measured using 20 Ω external series resistance to operate the device in short-circuit. Similarly, transient photovoltage was measured using 1 MΩ external series resistance to operate the device in open-circuit. The voltage dynamics on the resistors were recorded on a digital oscilloscope (Tektronix MDO3032). The perturbation red light source was set to a suitably low level to the white diodes array with light intensity equivalent to 100 mW cm^*–*2^ of a standard solar simulator.

### Data availability

The data that support the findings of this study are available from the corresponding author upon request.

## Additional information

**How to cite this article:** Bi, E. *et al*. Diffusion engineering of ions and charge carriers for stable efficient perovskite solar cells. *Nat. Commun.* **8,** 15330 doi: 10.1038/ncomms15330 (2017).

**Publisher's note**: Springer Nature remains neutral with regard to jurisdictional claims in published maps and institutional affiliations.

## Supplementary Material

Supplementary Information Supplementary Figures, Supplementary Tables, Supplementary Methods and Supplementary References
