# Supplementary material for: Diffusion engineering of ions and charge carriers for stable efficient perovskite solar cells
Source: Nat Commun. 2017 Jun 12;8:15330. doi: 10.1038/ncomms15330 (PMC5472713; doi:10.1038/ncomms15330)
Supplement: Supplementary Information — Supplementary Figures, Supplementary Tables, Supplementary Methods and Supplementary References [file ncomms15330-s1.pdf]

Supplementary methods

Chemicals:

All the chemicals were used as received, including PbI_2 (99%, Sigma-Aldrich), CH_3NH_3I (> 98%, Tokyo Chemical Industry Co., Japan), Titanium isopropoxide (99.999%, Sigma-Aldrich), nickel acetylacetonate (95%, Sigma-Aldrich), PCBM (99.5%, Lumtec Co., Taiwan). Magnesium acetatetetrahydrate (99%), lithium acetate (99%) and super dehydrated solvents of dimethylsulfoxide (DMSO), toluene, chlorobenzene, acetonitrile, methanol and ethanol, were all purchased from Wako Co., Japan. Ethanolamine and hydrogen peroxide aqueous solution (30% hydrogen peroxide) were of analytical-reagent grade and purchased from Sinopharm Chemical Reagent Co., Ltd (Shanghai, China).

Functionalized Graphene Synthesis:

Graphite (3 g) and $NaNO_3$ (1.5 g) were stirred together in a concentrated H_2SO_4 (69 mL, 98%) in an ice bath for 24 h. After this, under the condition of stirring and temperature not more than 20 °C, $KMnO_4$ (9 g) was added slowly. Then, the mixture was transferred into 35 °C water bath and vigorously stirred for 30 min. Subsequently, 138 mL of water was gradually added to the mixture and stirred for 30 min in the case of the temperature increased to 95±5 °C. Further, 213 mL of H_2O was added to dilute the mixture and moderate amount of H_2O_2 (30%) was added to neutralize the unreacted $KMnO_4$. Finally, the resulting brown color mixture was washed with diluted HCl (5%), ethanol, and warm water, and then dispersed in H_2O for 2 days ultrasonic irradiation.

The functionalized graphene was obtained from the nitrogen doping process[1]. The obtained GO samples were placed in the center of a tube furnace. After flowing NH_3 for about 10 min, the samples were places in a 300 °C furnace for 20 min and then heated to the reaction temperature to 700 °C. The reaction time was maintained for 2h inside the furnace before cooling. The samples were taken out of the tube reactor after the furnace temperature cooled to 50 °C. Finally, the graphene was washed by warm water and ethanol three times in order to purify the samples. The as-obtained graphene was dialyzed in the DI-water for 48 h. The dispersion was ultrasonicated for 10 hours and ultracentrifugated. After ultracentrifugation, the supernatant is extracted with a filter (0.2 μm caliber).

The graphene was sonicatied in chlorobenzene for 9 hours and graphene concentrations were controlled with respect to PCBM. The graphene with a 2 wt% and PCBM with the concentration of 40 mg mL^{-1} were co-dissolved in the chlorobenzene and stirred at 85°C for 12 hours under N_2 atmosphere.

Carbon Quantum Dots Synthesis:

In the synthesized experiment of carbon quantum dots, the 3 ml ethanolamine and 4.5 ml hydrogen peroxide aqueous solution (H_2O_2) was stirred at 250 °C and kept for 7 minutes. In the process of preparing carbon quantum dots, ethanolamine color changed into bright yellow just after adding H_2O_2, and then the color becomes dark finally. In this process, all the H_2O_2 have been reacted and water was volatilized. The obtained dark colloidal solid was added into 200 ml distilled water then the carbon quantum dots solution was obtained. The solution of carbon quantum dots was centrifuged at 14000 rpm for 30 min. The ultra-small carbon quantum dots products were obtained by the freeze drying process. Finally, carbon quantum dots products were re-dissolved in methanol solution with a 0.2 mg ml^{-1} concentration.

Perovskite precursor synthesis:

CH_3NH_3I was synthesized by reacting 24 mL of methylamine (33 wt. % in absolute ethanol, Sigma) and 10 mL of hydroiodic acid (57 wt% in water, Aldrich) in a 250 mL round-bottom flask at 0 °C for 2 h with stirring. The precipitate was recovered by putting the solution on a rotary evaporator and carefully removing the solvents at 50 °C. The yellowish raw product CH_3NH_3I was re-dissolved in 80 mL absolute ethanol and precipitate with the addition of 300 mL diethyl ether. After filtration, the step was repeated again. And the solid was collected and dried at 60 °C in a vacuum oven for 24 h.

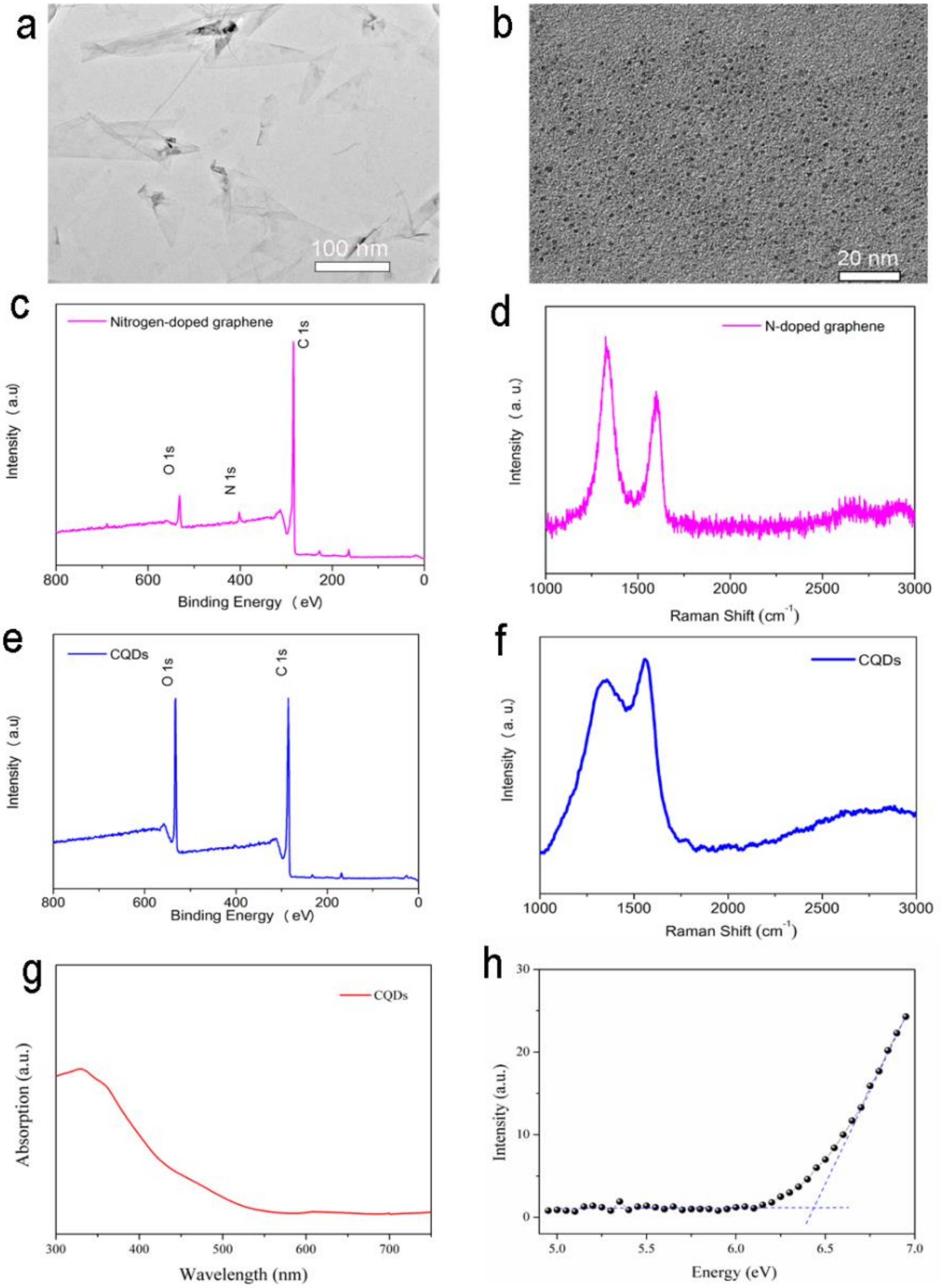

Supplementary Figure 1. Characterization of nanostructured carbon derivatives. Transmission electron microscopy (TEM) image of **a**, N-doped graphene and **b**, CQDs. The graphene size has a range of 100-200 nm; the carbon quantum dots have an average

size of about 2 nm. **c**, X-ray photoelectron spectroscopy (XPS) of graphene; **d**, Raman spectra of graphene. The XPS spectra confirms that the N element was doped in the graphene with a concentration of 4.62 %.[1, 2, 3] The Raman revealed that the graphitic structure of nitrogen doped graphene was almost completely restored as judged by I_D/I_G ratios.[4, 5] **e**, XPS of CQDs. The XPS spectrum reveals that carbon and oxygen are present at the surface of CQDs; **f**, Raman spectrum of the CQDs with excitation wavelength of 532 nm. The intensity ratio (I_D/I_G) obtained from two Raman peaks at 1352 cm^{-1} (D band) and 1574 cm^{-1} (G band) is ca. 0.82, suggesting a relatively high degree of graphitization of the CQDs.[6] **g**, The UV-Vis spectra of CQDs. **h**, the AC-3 UV photoelectron spectrometer spectrum of CQDs. HOMO levels of -6.42 eV were determined by AC-3 UV photoelectron spectrometer (Riken Keiki Co.). The conduction band edge value of -4.1 eV was obtained by adding the optical band gap of CQDs with 2.1 eV taken from the UV-Vis spectra.

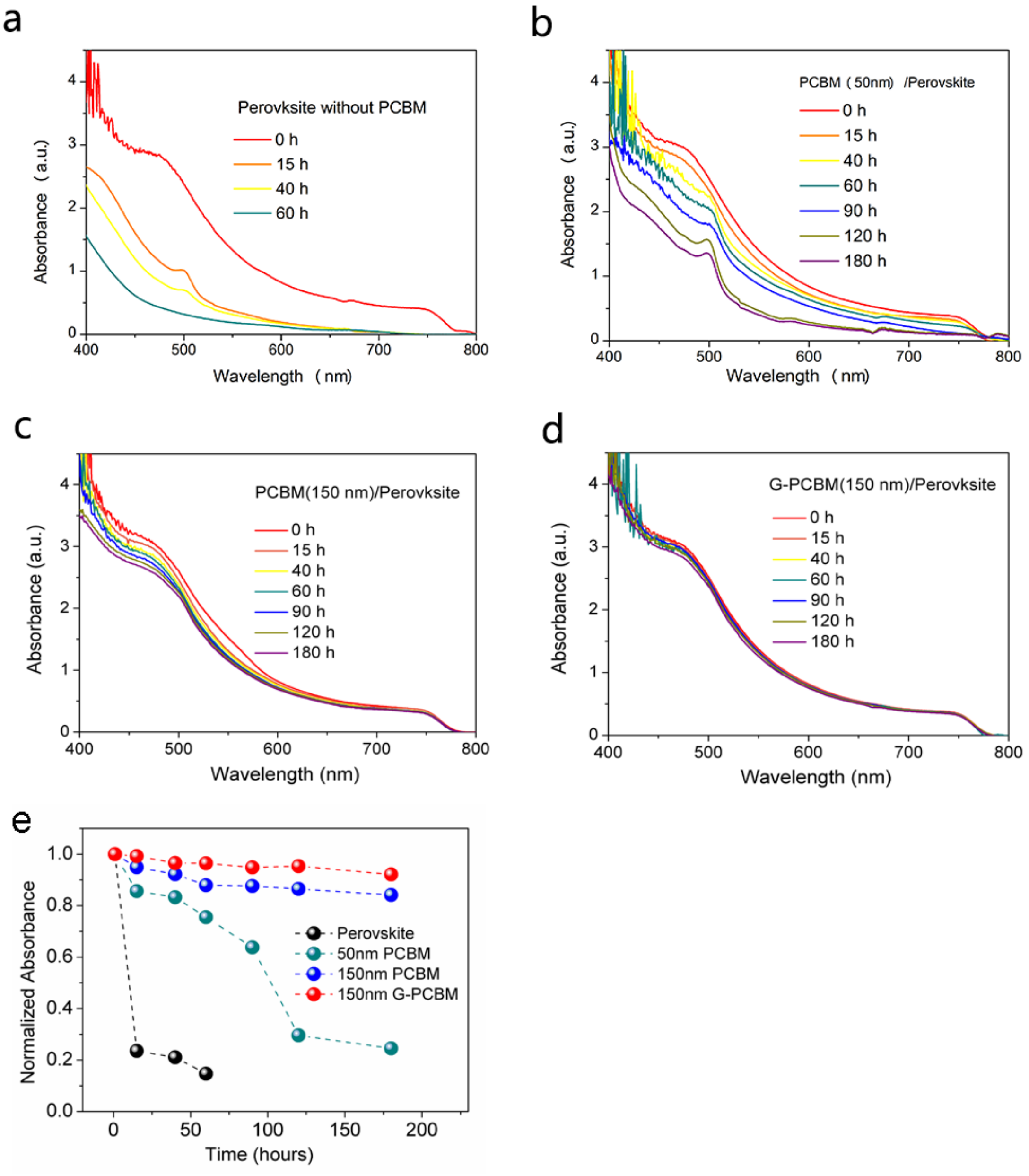

Supplementary Figure 2. Monitoring perovskite degradation by UV-Vis absorption spectroscopy of perovskite films ($MAPbI_3$, 350 nm). a, Perovskite film without PCBM; **b**, Perovskite film covered with 50 nm thick PCBM; **c**, Perovskite film with 150 nm thick PCBM; **d**, Perovskite film with 150 nm thick G-PCBM. **e,** The light absorbance at 600 nm (normalized by the initial values) as a function of time for the perovskite/ETL films. All the samples were made on NiMgLiO/FTO substrates; The samples were kept in dry N_2 and subjected to a temperature of 100 °C in the dark condition for 180 h.

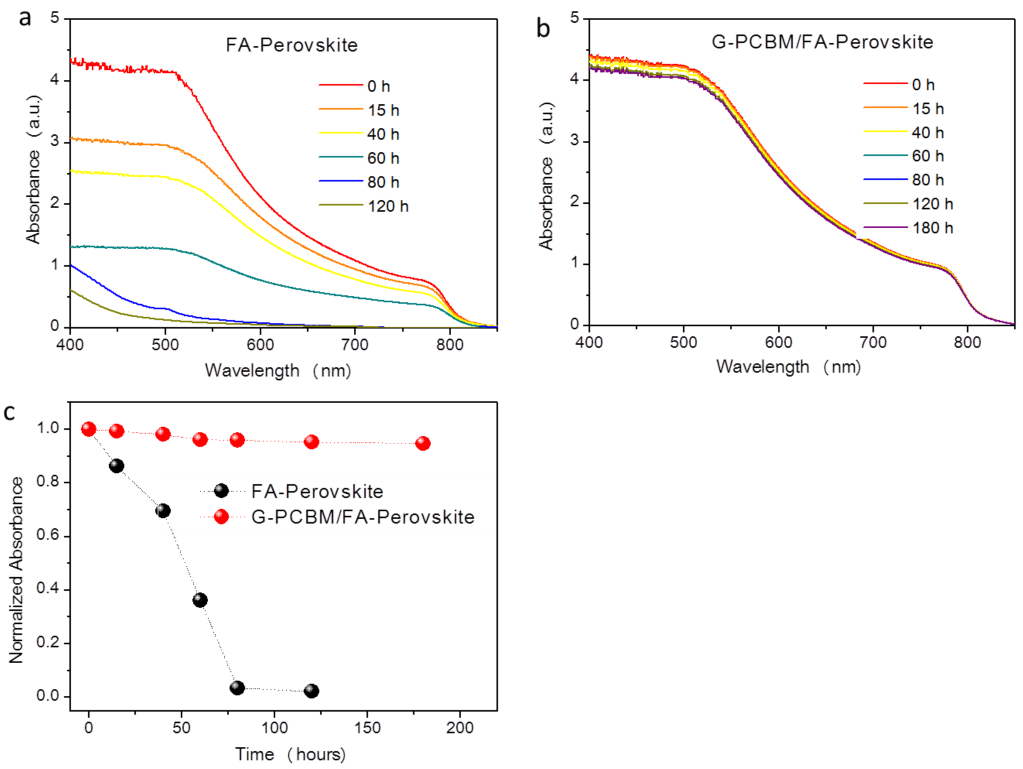

Supplementary Figure 3. Monitoring perovskite degradation by UV-Vis absorption spectroscopy of FA-Perovskite ($(FAPBI_3)_{0.85}(MAPbBr_3)_{0.15}$, 350 nm) films. a, Perovskite film without PCBM; **b**, Perovskite film covered with 150 nm thick G-PCBM; **c**, The light absorbance at 600 nm (normalized by the initial values) as a function of time. All the samples were made on NiMgLiO/FTO substrates; The samples were kept in dry N_2 and subjected to a temperature of 120 °C in the dark condition for 180 h.

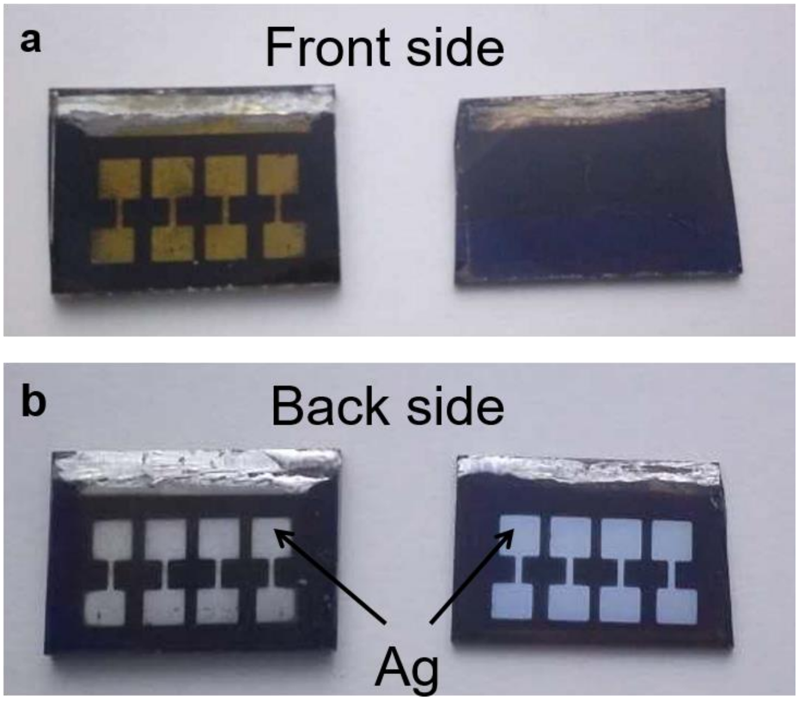

Supplementary Figure 4. Illustration of unsealed perovskite solar cells after aging test in a dry cabinet (<30 % humidity) in the dark for 50 days at 60 ^{o}C. The images are the **a**, front side and **b**, back side of cells with structure of perovskite/PCBM(150 nm)/CQDs/Ag (left) and perovskite/G-PCBM (150nm)/CQDs/Ag (right).

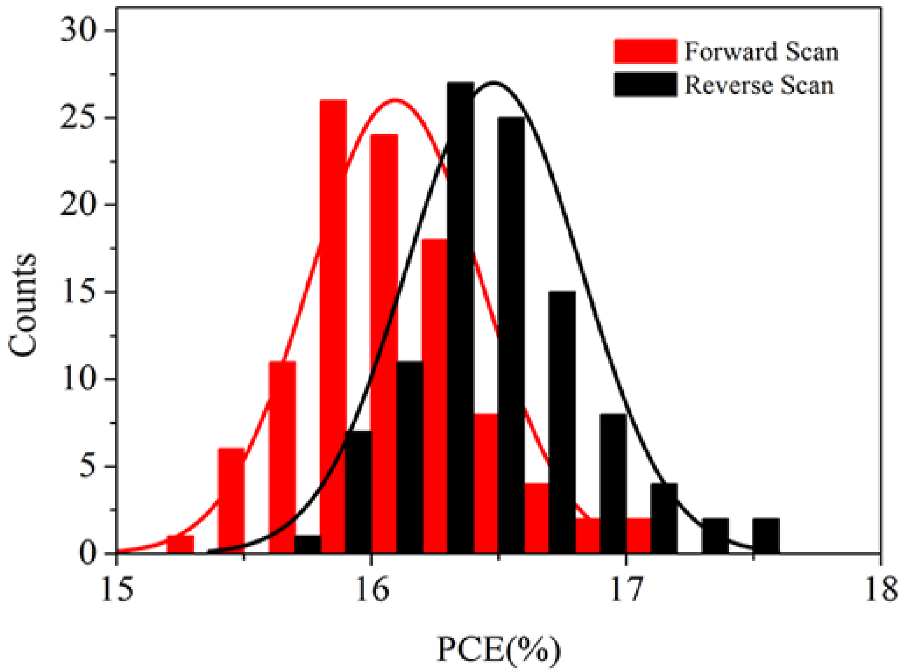

Supplementary Figure 5. The hysteresis effect observed in the present large area devices. A histogram comparing the difference of forward and reverse scan direction in the *PCE*s of the devices. The devices were measured under 100 mW cm^{-2} AM 1.5G illumination under forward scan (from short-circuit to open-circuit) and reverse scan (from open-circuit to short-circuit) at the step of 10 mV and delay time 50 ms. The device structure is FTO/NiMgLiO (20 nm)/ $MAPbI_3$ (350nm) /G-PCBM (150 nm)/CQDs (10 nm) /Ag (100 nm).

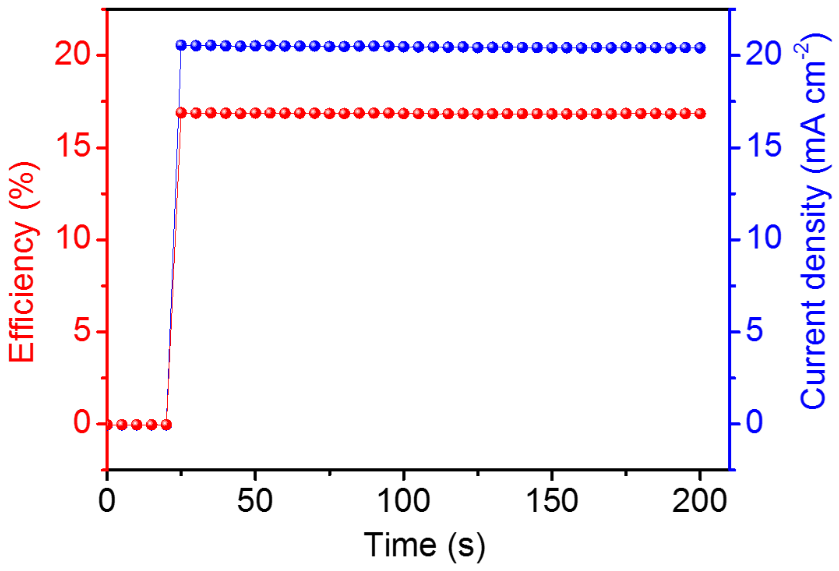

Supplementary Figure 6. Short-term stability of a device with CQDs/G-PCBM (150 nm). The device was measured at a bias of 0.82 V under 100 mW cm^{-2} AM 1.5G simulated solar light.

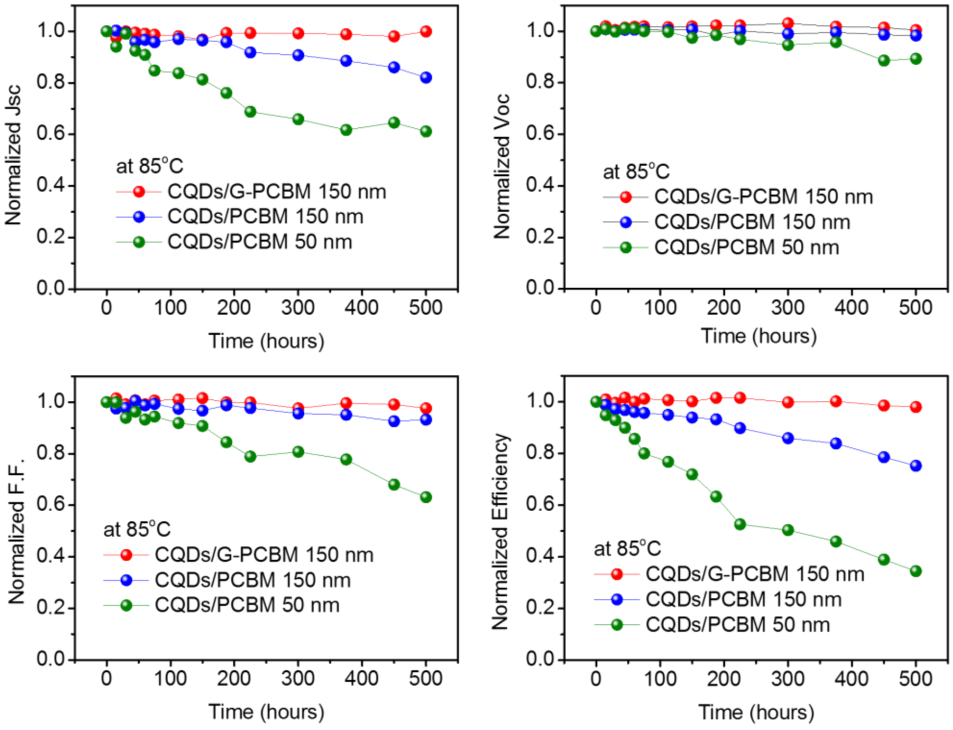

Supplementary Figure 7. Long-term stability of sealed cells. The devices were kept in thermal aging test at 85 ºC in an atmosphere with relative humidity of about 50 % and measured under AM 1.5G simulated solar light.

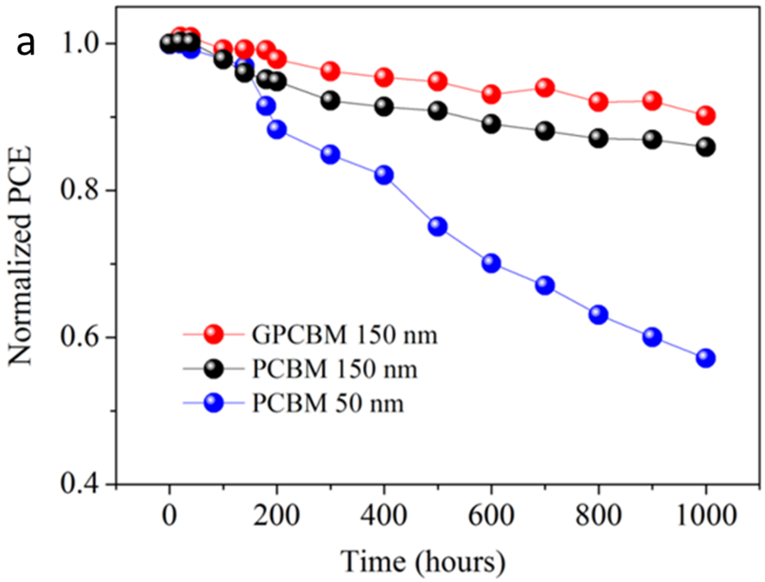

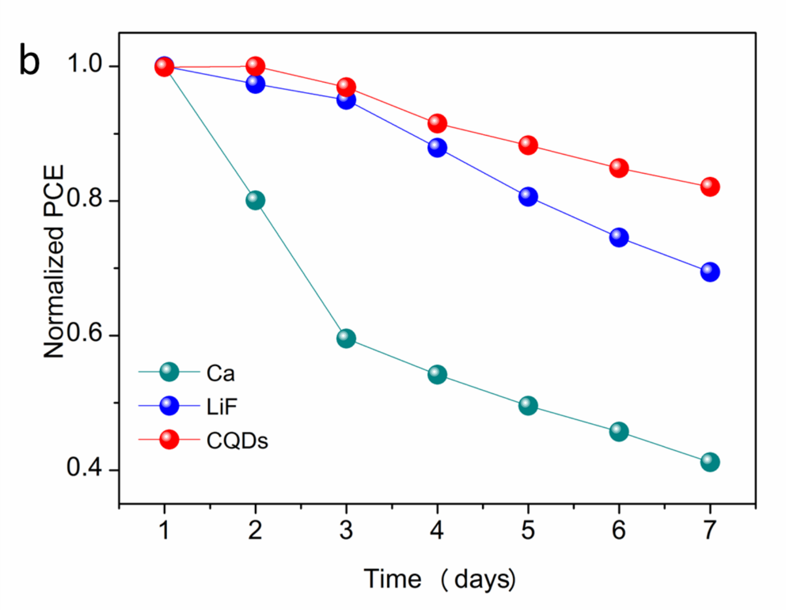

Supplementary Figure 8. Evaluation of unsealed device performance. a, devices with CQDs/G-PCBM (150 nm), CQDs/PCBM (150 nm) and CQDs/PCBM (50 nm), respectively. **b**, devices with Ca, LiF, and CQDs, respectively. The devices were kept in a dry cabinet (<30 % humidity) in the dark and measured in the ambient air under 100 mW cm^{-2} AM 1.5G simulated solar light.

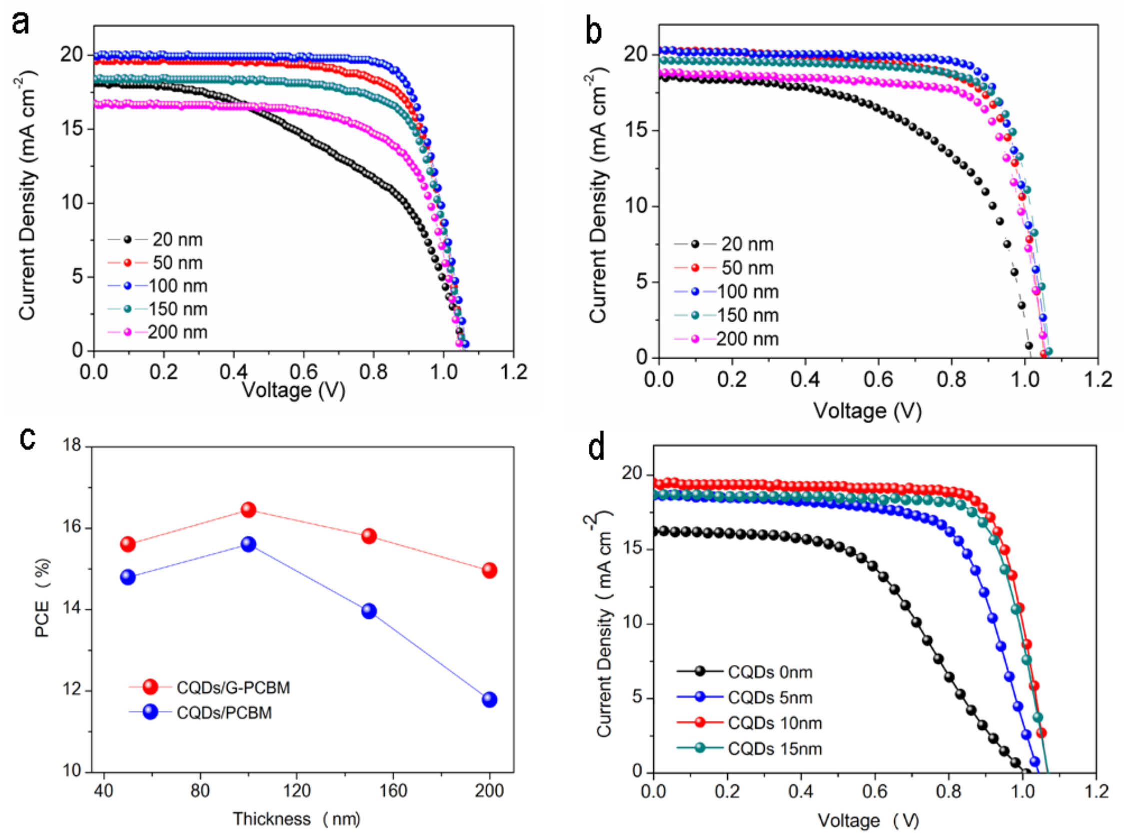

Supplementary Figure 9. Performance of device with different thickness of EEL. *J-V* characteristic of perovskite device (aperture area 1.02 cm^2) with different thickness of **a** PCBM and **b** G-PCBM, respectively. **c**, The PCEs for devices with varying thickness of CQDs/PCBM or CQDs/G-PCBM. **d,** The *J-V* curves for perovskite solar cells with varying thickness of CQDs.

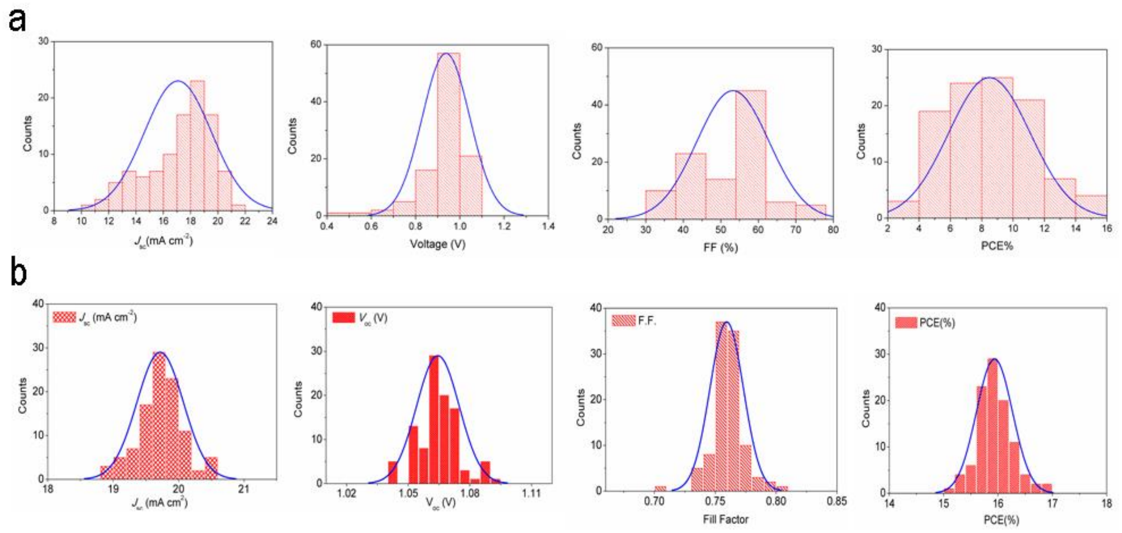

Supplementary Figure 10. a, The histograms for the performance metrics distribution of perovskite devices (aperture area 1.02 cm^2) using PCBM (50 nm). **b,** The histograms for devices with G-PCBM (150 nm). The devices were measured under 100 mW cm^{-2} AM 1.5G illumination under forward bias scan (from short-circuit to open-circuit) at the step of 10 mV and delay time 50 ms.

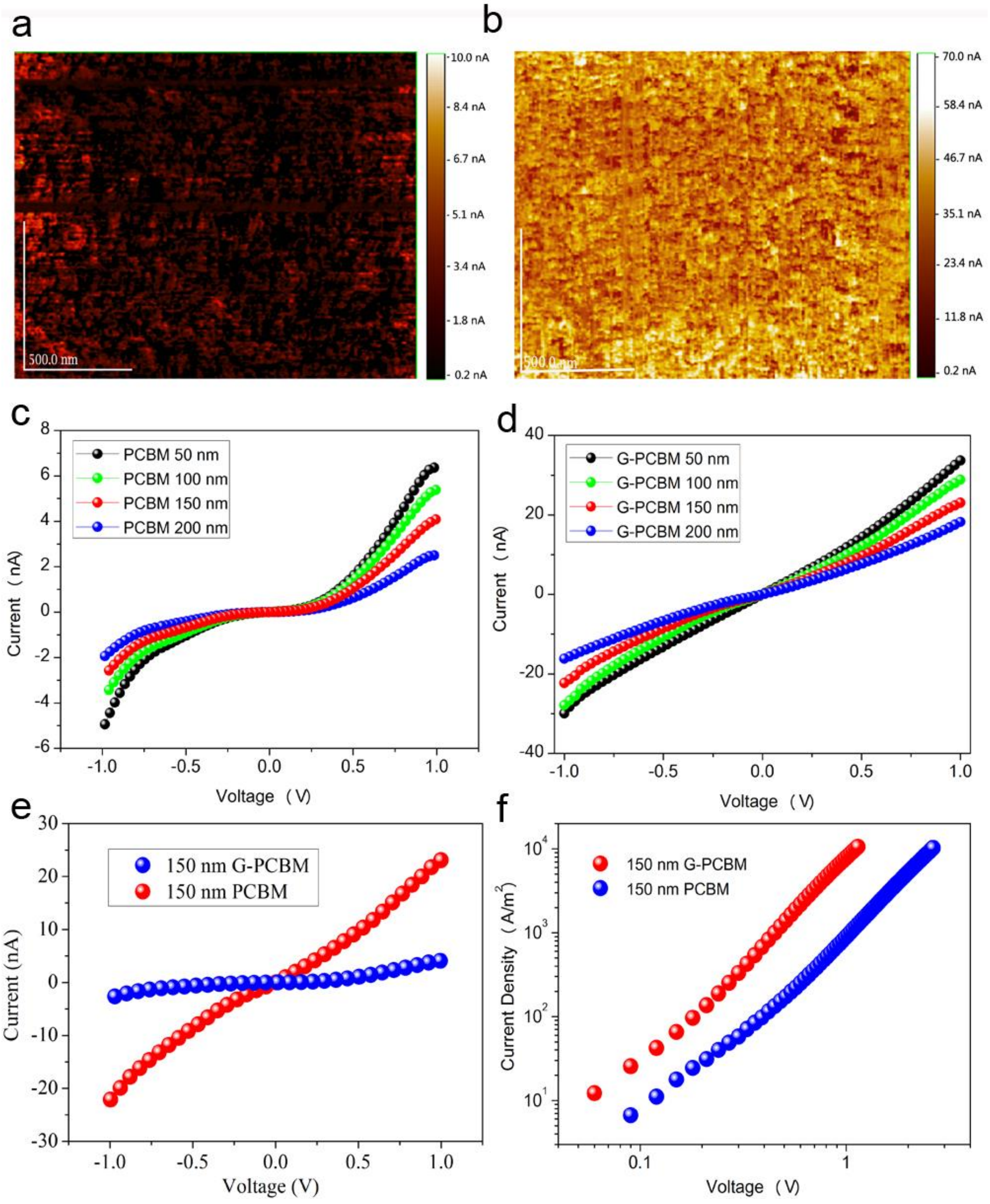

Supplementary Figure 11. Conductivity of PCBM and G-PCBM. a, conductivity mapping image of Scanning Probe Microscope for PCBM. **b,** The conductivity mapping image of G-PCBM films. Conductive *I-V* curves for **c**, G-PCBM films with different thickness on ITO substrate and **d**, PCBM films with different thickness on ITO substrate. **e**, Conductive *I-V* curves for 150 nm G-PCBM and PCBM films. **f**. The characteristics of space charge limited current for PCBM and G-PCBM film.

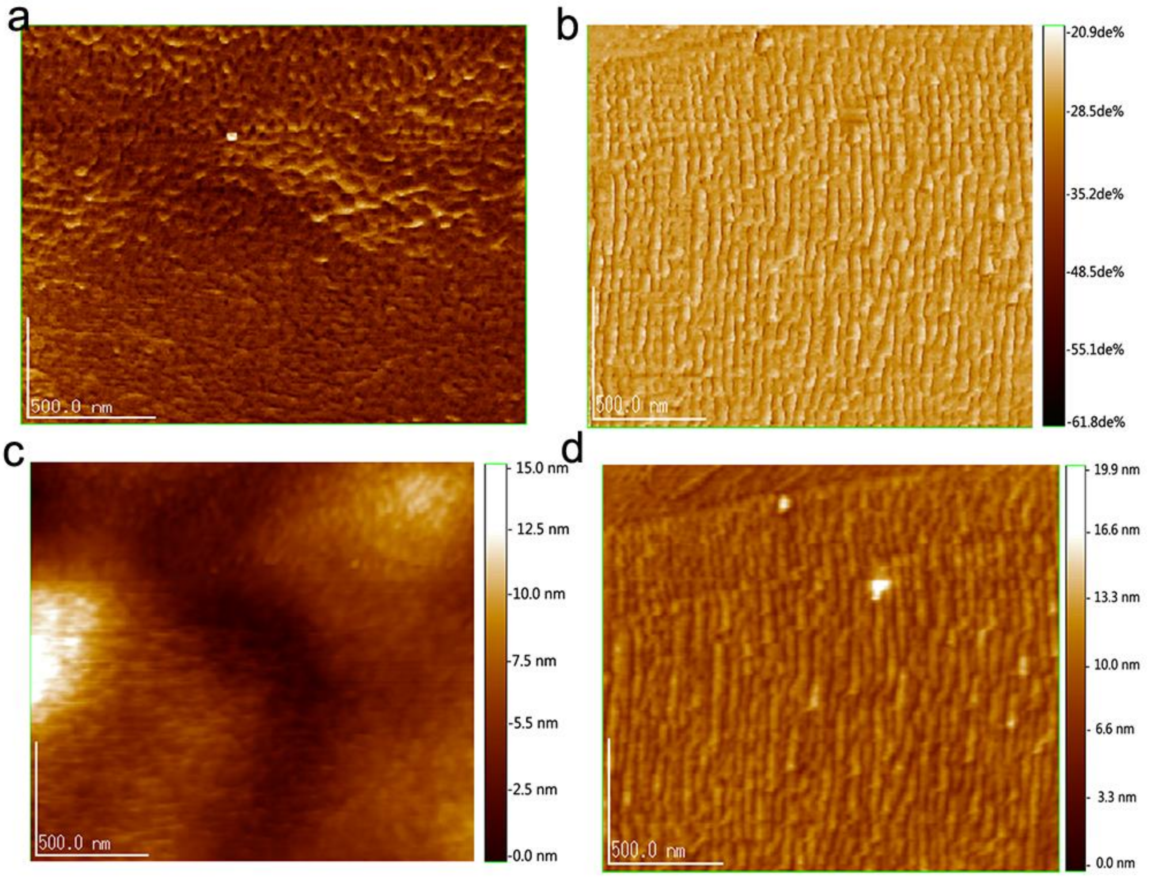

Supplementary Figure 12. Morphology of PCBM and G-PCBM films measured by atomic force microscopy (AFM). a-b, The AFM phase images of PCBM (left) and G-PCBM (right) films. **c-d**, The AFM topography images of PCBM (left) and G-PCBM (right) films.

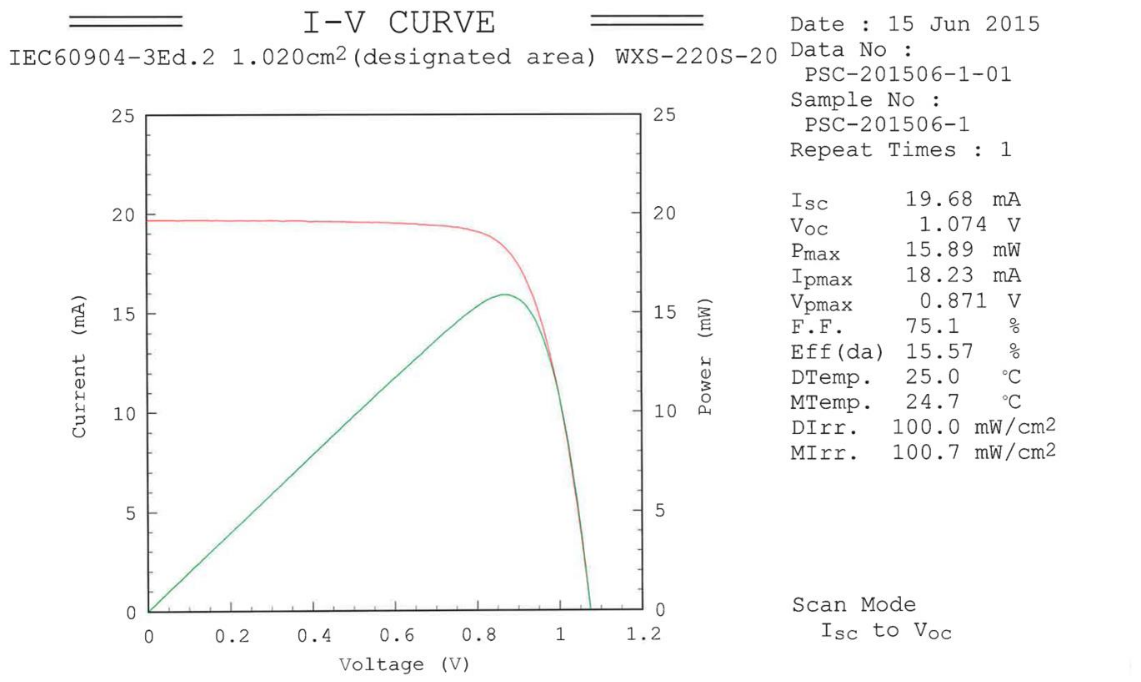

Supplementary Figure 13. The certified result from AIST with calibrated cell size of 1.02 cm^2.

Supplementary Table 1. Summary of device performance for perovskite device (aperture area 1.02 cm^2) with PCBM different thickness with forward scan at the step of 10 mV and the delay time 50 ms. The device structure is FTO/ NiMgLiO (20 nm)/ $MAPbI_3$ (350nm) /PCBM /CQDs (10 nm) /Ag (100 nm). The devices were measured under 100 mW cm^{-2} AM 1.5 G illumination.

PCBM	J_{SC} (mA cm^{-2})	V_{OC} (V)	*FF* (%)	*PCE* (%)
20 nm	18.01	1.061	46.7	8.92
50 nm	19.72	1.055	71.0	14.77
100 nm	19.92	1.066	74.0	15.72
150 nm	18.44	1.055	71.7	13.96
200 nm	16.66	1.048	67.5	11.79

Supplementary Table 2. Summary of device performance for perovskite device (aperture area 1.02 cm^2) with G-PCBM different thickness with forward scan at the step of 10 mV and the delay time 50 ms. The device structure is FTO/ NiMgLiO (20 nm)/ $MAPbI_3$ (350nm) /G-PCBM /CQDs (10 nm) /Ag (100 nm). The devices were measured under 100 mW cm^{-2} AM 1.5G illumination.

G-PCBM	J_{SC} (mA cm^{-2})	V_{OC} (V)	*FF* (%)	*PCE* (%)
20 nm	18.59	1.017	0.560	10.58
50 nm	19.94	1.053	0.728	15.30
100 nm	20.18	1.065	0.765	16.45
150 nm	19.69	1.068	0.751	15.80
200 nm	18.92	1.062	0.739	14.96

Supplementary Table 3. Summary of device performance for perovskite device (aperture area 1.02 cm^2) with CQDs different thickness with forward scan at the step of 10 mV and the delay time 50 ms. The device structure is FTO/NiMgLiO(20 nm)/ $MAPbI_3$ (350nm) /PCBM (100 nm)/QDs /Ag (100 nm). The devices was measured under 100 mW cm^{-2} AM 1.5 G illumination.

CQDs	J_{SC} (mA cm^{-2})	V_{OC} (V)	*FF* (%)	*PCE* (%)
0 nm	16.24	1.008	0.494	8.09
5 nm	18.63	1.023	0.662	12.62
10 nm	19.38	1.068	0.765	15.83
15 nm	18.69	1.069	0.752	15.03

Supplementary Table 4. Summary of device performance for perovskite device (aperture area 1.02 cm^2) with different electron extraction layers (EEL). The devices were measured under 100 mW cm^{-2} AM 1.5 G illumination.

EEL		J_{SC} (mA cm^{-2})	V_{OC} (V)	*FF* (%)	*PCE* (%)
PCBM	average	16.2	1.01	0.494	8.09
	best	18.2	1.03	0.563	10.5
G-PCBM	average	17.6	1.02	0.542	9.71
	best	19.1	1.05	0.612	12.3
CQDs/PCBM	average	18.4	1.05	0.717	13.9
	best	19.4	1.06	0.734	15.1
CQDs/G-PCBM	average	19.7	1.07	0.751	15.8
	best	20.6	1.08	0.766	17.0

Note: the thickness of both PCBM and G-PCBM is 150 nm; the data were analysed from a total of 40 cells for each group.

References

1. HoonáJun G, HwanáJin S, HyunáKim B, HyungáHong S. Enhanced conduction and charge-selectivity by N-doped graphene flakes in the active layer of bulk-heterojunction organic solar cells. *Energy & Environmental Science* 2013, **6**(10)**:** 3000-3006.

2. Lu L, Xu T, Chen W, Lee JM, Luo Z, Jung IH*, et al.* The role of N-doped multiwall carbon nanotubes in achieving highly efficient polymer bulk heterojunction solar cells. *Nano Letters* 2013, **13**(6)**:** 2365-2369.

3. Lee JM, Park JS, Lee SH, Kim H, Yoo S, Kim SO. Selective Electron‐ or Hole‐Transport Enhancement in Bulk‐Heterojunction Organic Solar Cells with N‐or B‐Doped Carbon Nanotubes. *Advanced Materials* 2011, **23**(5)**:** 629-633.

4. Parvez K, Yang S, Hernandez Y, Winter A, Turchanin A, Feng X*, et al.* Nitrogen-doped graphene and its iron-based composite as efficient electrocatalysts for oxygen reduction reaction. *ACS Nano* 2012, **6**(11)**:** 9541-9550.

5. Ju MJ, Kim JC, Choi H-J, Choi IT, Kim SG, Lim K*, et al.* N-doped graphene nanoplatelets as superior metal-free counter electrodes for organic dye-sensitized solar cells. *ACS Nano* 2013, **7**(6)**:** 5243-5250.

6. Li H, He X, Kang Z, Huang H, Liu Y, Liu J*, et al.* Water‐Soluble Fluorescent Carbon Quantum Dots and Photocatalyst Design. *Angewandte Chemie International Edition* 2010, **49**(26)**:** 4430-4434.
